# Understanding Crosstalk Between Phosphate and Immune-Related Signaling in Rice and *Arabidopsis* Through Live Imaging of Phosphate Levels

**DOI:** 10.3390/plants14213334

**Published:** 2025-10-31

**Authors:** Mani Deepika Mallavarapu, María Ribaya, Beatriz Val-Torregrosa, Blanca San Segundo

**Affiliations:** 1 Centre for Research in Agricultural Genomics (CRAG), Campus Universitat Autónoma de Barcelona, C/de la Vall Moronta s/n, Bellaterra (Cerdanyola del Vallés), 08193 Barcelona, Spain; mani.mallavarapu@cragenomica.es (M.D.M.); mribaya.mu@gmail.com (M.R.); beatorregrosa@gmail.com (B.V.-T.); 2 Consejo Superior de Investigaciones Científicas (CSIC), 08001 Barcelona, Spain

**Keywords:** FRET biosensor, phosphate, cytosolic Pi, FLIPPi, *Arabidopsis*, rice, phosphite, nutrient sensing, plant immunity, live imaging, nutrient-immunity crosstalk

## Abstract

Phosphate (Pi) is a vital macronutrient for plant growth and development, and precise monitoring of its cellular dynamics is essential to understanding Pi homeostasis and its interaction with stress responses. Genetically encoded FRET-based biosensors such as FLIPPi enable real-time, non-invasive visualization of cytosolic Pi levels in living tissues. In this study, *Arabidopsis* and rice lines expressing a FLIPPi biosensor were used to monitor cytosolic Pi dynamics in root epidermal cells. Sensor functionality was confirmed by measuring FRET responses to graded Pi supplies, revealing a consistent reduction in FRET ratios with increasing Pi concentrations, reflecting elevated cytosolic Pi levels. Comparisons with a Pi-insensitive FLIPPi variant confirmed the specificity of the observed changes. Furthermore, live imaging demonstrated rapid and dynamic alterations in cytosolic Pi upon treatment with defense-related hormones and elicitors of immune responses supporting a link between Pi signaling and plant immunity. Finally, the application of phosphite, an analog of Pi, altered Pi dynamics in both *Arabidopsis* and rice, suggesting an interference with Pi accumulation. Collectively, our findings establish FLIPPi as a reliable tool for in vivo monitoring of Pi in *Arabidopsis* and rice plants, the model systems for studies in dicotyledonous and monocotyledonous species, respectively.

## 1. Introduction

Phosphorus (P) is an essential macronutrient necessary for numerous physiological processes in plants, including energy metabolism (ATP synthesis), signal transduction pathways, nucleic acid synthesis, and membrane integrity via phospholipid formation [1]. Plants predominantly absorb P through the roots in the form of inorganic phosphate (Pi). However, the bioavailability of Pi in soil is often severely limited due to its inherent low solubility and strong affinity for soil minerals and organic matter, leading to its immobilization into forms that plants cannot readily absorb [2]. Consequently, plants have developed complex adaptive strategies to optimize Pi acquisition under conditions of varying phosphate availability. To cope with Pi deficiency and maintain its homeostasis, plants initiate multifaceted morphological, physiological, and molecular adaptations [3].

Morphological adaptations are particularly prominent in root architecture alterations, such as the elongation of primary roots and enhanced lateral root and hair proliferation, which collectively enhance soil exploration and surface area for Pi uptake [4]. Physiologically, plants enhance the exudation of organic acids, phosphatases, and other enzymes into the rhizosphere, which solubilize Pi from insoluble complexes, thereby improving its availability to root systems. Molecularly, phosphate starvation triggers the extensive reprogramming of gene expression, prominently involving the upregulation of high-affinity phosphate transporters (PHT1 family). Under phosphate limitation, plants activate a complex regulatory network that enhances Pi uptake and optimizes internal Pi utilization to maintain Pi homeostasis, termed as phosphate starvation responses [5,6,7]. Conversely, excessive phosphate, often resulting from the overuse of phosphate fertilizers in agriculture, can adversely affect plant growth due to toxicity or by inducing nutrient imbalance.

In addition, alterations in Pi levels have been demonstrated to influence plant immune responses against pathogen infection, including defense-related hormone signaling pathways [8,9,10,11,12]. For instance, alterations in Pi levels were shown to modulate jasmonic acid (JA) and salicylic acid (SA) content in the leaves of *Arabidopsis* plants which, in turn, has an effect on the expression of defense-related genes [13]. Pathogen infection and treatment with molecules that stimulate the plant’s immune response (generally termed as elicitors) can also interfere with Pi signaling pathways and the expression of Pi transporter genes in *Arabidopsis* and rice plants [9,10,12]. Thus, a nuanced understanding of Pi homeostasis and its intricate interactions with hormonal signaling and immunity is critical for sustainable agriculture practices.

It is also known that phosphite (Phi, PO_3_^3−^), a reduced analog of phosphate (Pi, PO_4_^3−^), can be taken up by plant roots through Pi transporters, but cannot be metabolized by plants [14,15]. Phi has been shown to alter Pi levels and enhance resistance to pathogen infection in plants [16,17,18]. For example, the treatment of *Arabidopsis* plants with Phi was reported to confer protection against *Hyaloperonospora arabidopsidis* via SA-dependent defense responses, reflecting nutrient–immunity crosstalk [19]. A better understanding of the interactions between Pi and Phi signaling pathways in both biological processes, Pi nutrition and immunity, is crucial for optimizing crop performance and plant health.

Traditional methods used for the measurement of Pi content in plant tissues, such as spectrophotometric methods, inductively coupled plasma mass spectrometry (ICP-MS), or 31P-NMR spectroscopy, involve the destruction of the tissue’s cell integrity and preparation of plant extracts. Recent advances in live-cell imaging technologies using biosensors have significantly enhanced our ability to study nutrient dynamics at unprecedented spatial and temporal resolutions. Among these technologies, Förster resonance energy transfer (FRET) has emerged as a powerful tool for visualizing molecular interactions and biochemical processes within living cells [20]. FRET is based on the non-radiative energy transfer between two fluorescent proteins (donor and acceptor fluorophores) positioned in close proximity (ranging at about 1–10 nm). This efficiency of energy transfer depends strongly on the spatial orientation and distance between the fluorophores, making FRET exceptionally sensitive to conformational changes and molecular interactions in real time [21,22].

Building on the principles of FRET, genetically encoded biosensors such as the fluorescent indicator protein for phosphate (FLIPPi) have been engineered to dynamically monitor intracellular Pi levels. FLIPPi consists of a chimeric construct in which a bacterial periplasmic phosphate-binding protein (PiBP) from *Synechococcus* sp. (strain A, ORF01723) is fused to enhanced cyan fluorescent protein (eCFP; donor) and a yellow fluorescent protein, circularly permuted Venus (cpVenus; acceptor) [23]. When phosphate molecules bind to the PiBP, it undergoes a conformational change that modifies the relative orientation of eCFP and YFP, leading to detectable shifts in FRET efficiency, measurable by fluorescence microscopy [24]. FLIPPi sensors offer significant advantages compared to traditional measurement methods that fail to provide real-time monitoring and require tissue disruption, making them unsuitable for investigating rapid or localized phosphate dynamics in vivo. FLIPPi, in contrast, enables non-invasive, real-time, and high-resolution imaging of Pi dynamics at the cellular and subcellular scales. However, although FLIPPi and other FRET-based biosensors ensure accurate and reproducible measurements for Pi quantification, their application in plants presents certain challenges and limitations. Additionally, sensor accuracy and sensitivity may be influenced by intrinsic cellular factors such as autofluorescence or non-specific interactions between the sensor proteins and cellular components, which may alter their properties or subcellular localization [23,25]. Thus, rigorous calibration, control experiments employing phosphate-insensitive sensor variants, and validation steps are necessary for accurate data interpretation in distinct plant species. FLIPPi sensors have been previously used to investigate the accumulation of Pi in the roots of *Arabidopsis thaliana* under Pi starvation conditions [23,26,27]. Despite these advances, the application of FLIPPi-based imaging to crop plants for studying Pi content and homeostasis remains in its infancy. Extending the use of such genetically encoded biosensors beyond *Arabidopsis* is pivotal for advancing our understanding of Pi dynamics in agriculturally relevant systems to advance timely monitoring and solutions. Pi deficiency is one of the major factors limiting crop productivity worldwide, and precise monitoring of Pi status at the cellular level could provide valuable insights into plant adaptation and nutrient efficiency mechanisms.

Rice, *Oryza sativa*, a globally important staple crop, often suffers from inadequate Pi availability in soils. On one hand, growing rice in low-Pi soil leads to stunted growth and reduced yield. On the other hand, the overuse of fertilizers to circumvent the limited bioavailability of Pi has led to a scenario of excessive soil P in rice fields. On this basis, employing FLIPPi sensors in rice therefore offers an unprecedented opportunity to visualize phosphate dynamics in a monocot system, enabling a comparative analysis with *Arabidopsis* and supporting the development of strategies to improve phosphate-use efficiency and crop resilience.

In this study, we employed a FRET-based Pi biosensor, FLIPPi5.3m, designed for in vivo imaging of cytosolic Pi, to investigate Pi dynamics in both *Arabidopsis thaliana* and *Oryza sativa*. Transgenic sensor lines were optimized for the live-cell imaging of root epidermal tissues, enabling non-invasive and real-time quantification of cytosolic Pi levels. Using these lines, we analyzed FRET responses to varying external Pi supplies, as well as to treatments with defense-related hormones and elicitors of immune responses, to evaluate dynamic changes in intracellular Pi. Additionally, we examined the effect of phosphite (Phi) on cytosolic Pi content to assess potential interference with phosphate homeostasis. Together, these experiments establish the FLIPPi system as a robust approach for visualizing phosphate dynamics and nutrient–immunity interactions in both dicot and monocot model systems, offering valuable insights for improving phosphate-use efficiency and crop stress resilience, expanding the scope of sustainable rice production systems.

## 2. Results

### 2.1. Generation and Validation of FLIPPi Sensor Lines in Arabidopsis and Rice for Live Imaging of Cytosolic Pi

Transgenic *Arabidopsis* lines constitutively expressing the cpFLIPPi5.3m biosensor were used to monitor cytosolic Pi dynamics in vivo. This cpFLIPPi5.3m variant was specifically designed for in vivo imaging of cytosolic Pi in *Arabidopsis* plants [23,26]. As controls, transgenic *Arabidopsis* lines expressing the Pi-insensitive variant cpFLIPPi-Null were assayed. Furthermore, in this work we generated stable transgenic rice (*Oryza sativa* cv. Tainung 67) lines constitutively expressing either the cpFLIPPi5.3m or cpFLIPPi-Null gene, thereby extending the FLIPPi biosensor application to a monocot crop species. Expression of the *PiBP* transgene in rice was confirmed by RT-qPCR (Figure S1).

**Figure 3:**
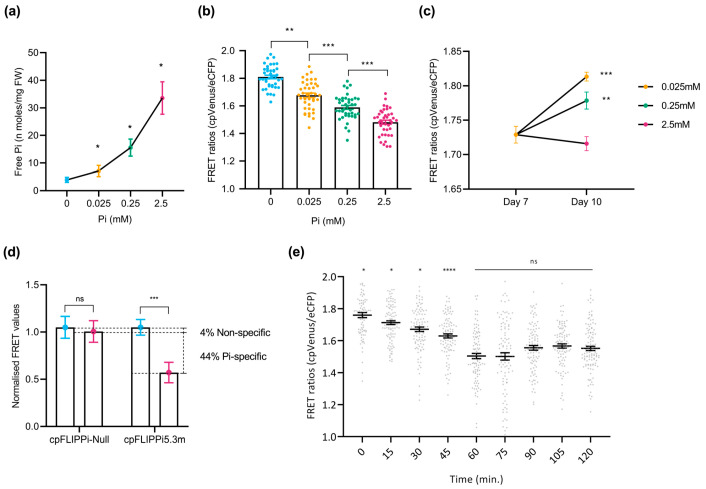
Live imaging of cytosolic Pi in the roots of rice plants in response to changes in Pi supply. FRET values were calculated from root epidermal cells in the transition–elongation region. Three independent experiments were carried out with similar results. The plots show data points of ratios of 10 readings taken per plant, with 10 plants per condition, and the error bars represent the ±SEM. Statistical significance of the data was analyzed using the one-way ANOVA test with a *p* value < 0.001 and multiple comparisons test within timepoints, and the significance compared to preceding data is indicated by * *p* ≤ 0.05, ** *p* ≤ 0.01, *** *p* ≤ 0.001, ****, *p* ≤ 0.0001, and ns: non-significant. Details of growth conditions and treatments in rice are shown in Table S2. (**a**) Spectrophotometric quantification of Pi concentration in roots of cpFLIPPi5.m rice plants grown under increasing Pi concentrations. (**b**) FRET ratios (cpVenus/eCFP) of roots from cpFLIPPi5.3m rice plants grown in ½ MS medium for first 7 days and then subjected to treatment with the indicated Pi concentrations for 3 days. (**c**) FRET ratios of cpFLIPPi5.3m rice roots grown in ½ MS medium measured at day 7, compared to measurements taken at day 10 after treatment in modified Hoagland medium supplemented with different Pi concentrations for 3 days. (**d**) FRET values of cpFLIPPi5.3m and cpFLIPPi-null plants (P_0_ and P_2.5_ conditions, normalized to P_0_). (**e**) Temporal dynamics of cytosolic Pi content during changes in Pi supply. FRET values of 10-day-old cpFLIPPi5.3m seedlings conditioned in low Pi (0.025 mM Pi) and supplied with 2.5 mM Pi solution were measured every 15 min over a period of 120 min. For each timepoint, at least 10 independent seedlings were analyzed. A multiple comparisons test was used within timepoints, and the significance compared to the preceding timepoint is indicated.

To monitor the phenotypic responses of *Arabidopsis* and rice cpFLIPPi5.3m plants to increasing Pi regimes, cpFLIPPi5.3m *Arabidopsis* seedlings were grown in half-strength MS medium for seven days, followed by seven more days in the modified Hoagland media supplemented with 0 mM Pi (no Pi was added to the growing medium), 0.025 mM Pi, 0.25 mM Pi, and 2.5 mM Pi (from now on referred to as P_0_, P_0.025_, P_0.25,_ and P_2.5_, respectively). At the root level, increasing Pi supply resulted in a continuous increase in *Arabidopsis* root length across the tested Pi concentrations (Figure S2a), supporting that plants respond to Pi treatment. In rice, the cpFLIPPi5.3m seedlings were grown in half-strength MS medium for seven days and then transferred to media at P_0_, P_0.025_, P_0.25,_ or P_2.5_ conditions for three more days. No visible morphological changes were observed in the root system of rice plants during the Pi treatment period (Figure S2b). Presumably, longer periods of treatment with Pi might be needed to induce visible morphological changes in the rice root system. However, the Pi content of these rice roots increased with increasing the Pi concentration (as shown below in Figure 3a), suggesting that the rice plants responded to the Pi treatment as desired.

**Figure 1:**
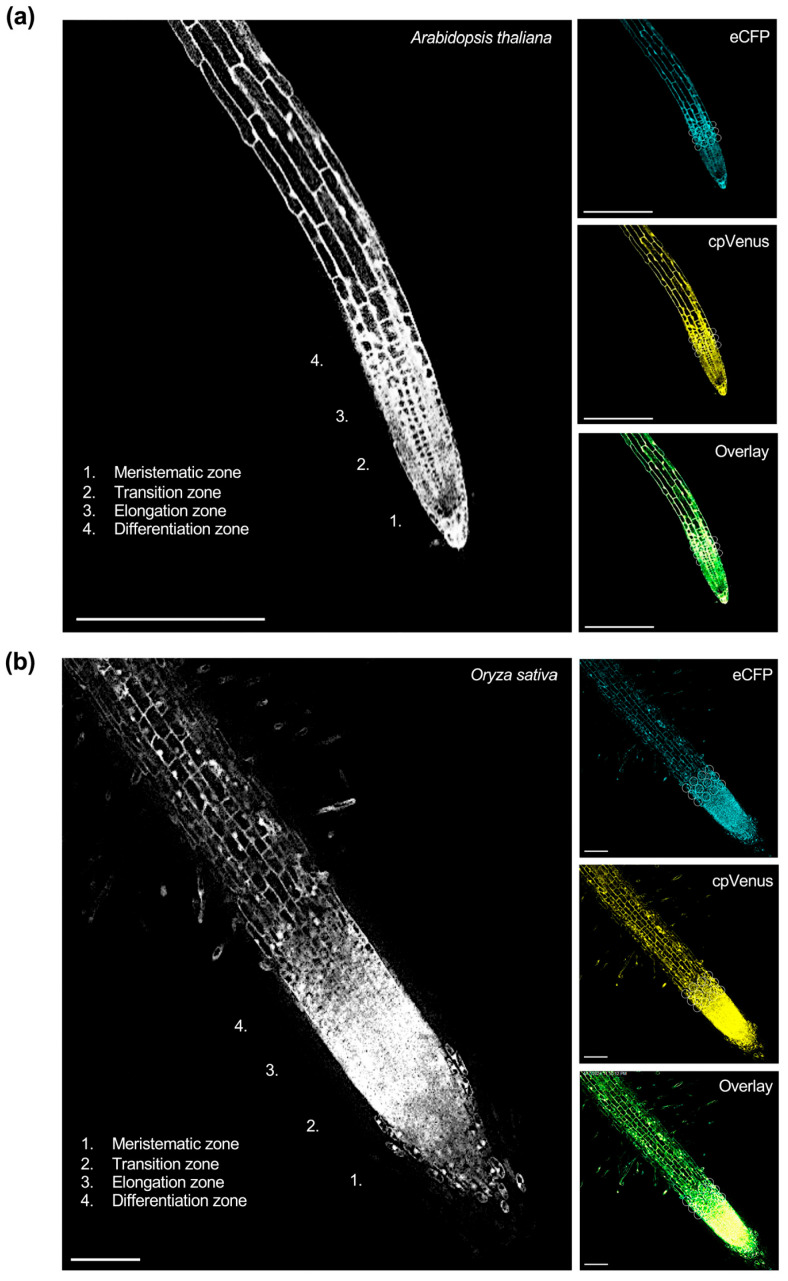
Live imaging of cytosolic Pi in the roots of T3 homozygous lines of (**a**) *Arabidopsis* and (**b**) rice expressing cpFLIPPi5.3m. FRET ratios were quantified in epidermal cells of the transition–elongation zone, with representative regions of interest (ROIs) for FRET measurements indicated by circles. The corresponding right panels show images captured in the eCFP channel (483/32 nm), cpVenus channel (542/27 nm), and the merged overlay of eCFP and cpVenus signals. Images shown represent single optical sections acquired by confocal laser scanning microscopy. (**a**) Confocal image (20×) of a primary root from cpFLIPPi5.3m *Arabidopsis* seedlings grown for 7 days in half-strength MS media followed by 7 days of treatment with 0.025 mM Pi. Scale bars, 250 µm. (**b**) Confocal image (20×) of a primary root from cpFLIPPI5.3 rice seedlings that had been grown for 7 days in ½ MS media, followed by 3 days of treatment with 0.025 mM Pi. Scale bars represent 100 µm.

To visualize these responses at the cellular scale, representative confocal fluorescence images of the cpFLIPPi5.3m-transformed *Arabidopsis* and rice root epidermal cells are shown in Figure 1, illustrating eCFP and cpVenus channel emissions, the corresponding FRET ratios, and merged overlays.

### 2.2. Pi Treatment Increases Cytosolic Pi Levels in Arabidopsis Roots Corresponding to Decreasing FRET Emission

**Figure 2:**
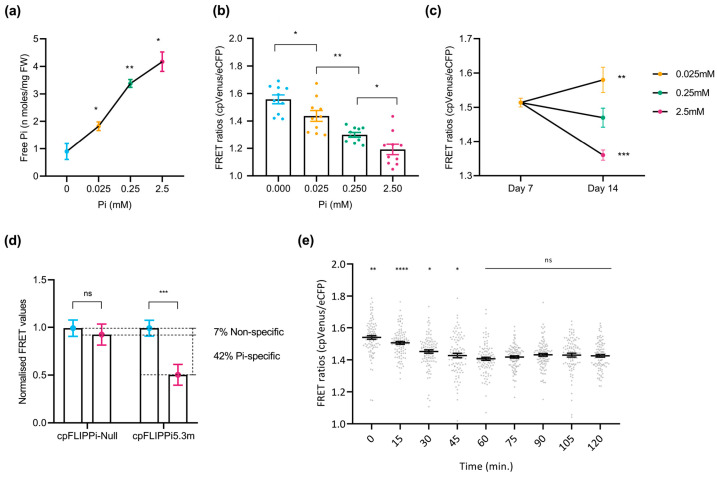
Live imaging of cytosolic Pi in *Arabidopsis* roots in response to changes in Pi supply. FRET values were calculated from root epidermal cells in the transition–elongation region. Three independent experiments were carried out with similar results, with 10 seedlings per condition, 10 readings per plant, and the error bars represent the ±SEM. Statistical significance of the data was analyzed using the one-way ANOVA test with a *p* value < 0.001 and a multiple comparisons test within timepoints, and the significance compared to preceding data is indicated by * *p* ≤ 0.05, ** *p* ≤ 0.01, *** *p* ≤ 0.001, **** *p* ≤ 0.0001, and ns: non-significant. Details on growth conditions and treatments in *Arabidopsis* are shown in Table S1. (**a**) Spectrophotometric quantification of Pi concentration in roots of cpFLIPPi5.m *Arabidopsis* plants grown under increasing Pi concentrations. (**b**) FRET ratios (cpVenus/eCFP) of cpFLIPPi5.3m *Arabidopsis* roots grown in ½ MS media for the first 7 days, followed by 7 days of treatment in modified Hoagland media with different Pi concentrations. (**c**) FRET ratios of cpFLIPPi5.3m *Arabidopsis* roots from ½ MS media at day 7, compared to varying Pi treatments at day 14. (**d**) Normalized FRET ratios of cpFLIPPi5.3m and cpFLIPPi-null plants (grown in P_0_ and P_2.5_ conditions, normalized to P_0_). (**e**) Temporal dynamics of cytosolic Pi content during changes in Pi supply with FRET values of cpFLIPPi5.3m *Arabidopsis* plants that were conditioned in low Pi (0.025 mM Pi) for 7 days and supplied with 2.5 mM Pi solution on the slide setup. FRET values were recorded every 15 min for 120 min. A multiple comparisons test was used within timepoints, and the significance was compared to the preceding timepoint.

To assess the changes in cytosolic Pi levels in response to the Pi treatments, root free Pi content was initially quantified spectrophotometrically in whole roots. A clear increase in Pi content with increasing Pi supply confirmed the physiological responsiveness of cpFLIPPi5.3m *Arabidopsis* plants to Pi availability (Figure 2a). Changes in FRET ratios, calculated as cpVenus/eCFP emission intensities, were analyzed in these cpFLIPPi5.3m lines. Consistent with the mechanism of FLIPPi biosensors, in which the binding of Pi molecules to the PiBP sensor reduces the FRET emission by cpVenus, the cpFLIPPi5.3m sensor lines exposed to increasing Pi concentrations displayed a significant reduction in FRET ratios (cpVenus/eCFP) (Figure 2b).

To further confirm that these changes are associated with external Pi treatments, FRET ratios were compared between seedlings at day 7 (grown in half-strength MS medium) and after 7 additional days under defined Pi regimes (P_0.025_, P_0.25_, and P_2.5_). As expected, seedlings grown at lower Pi concentrations (e.g., P_0.025_) exhibited higher FRET ratios, whereas those supplied with higher concentrations of Pi (e.g., P_2.5_) showed markedly reduced ratios (Figure 2c). This observed reduction in FRET ratios aligns with an increase in cytosolic Pi content in response to increasing Pi supply, and vice versa, thus confirming that the biosensor responds dynamically to changes in cytosolic Pi levels.

Furthermore, to validate the specificity of the FRET signal to Pi, we compared the FRET ratios of Pi-responsive cpFLIPPi5.3m and Pi-insensitive cpFLIPPi-Null plants grown without Pi (P_0_) and with Pi (P_2.5_). Notably, the cpFLIPPi5.3m sensor plants exhibited a Pi-dependent reduction in FRET ratios, as in these plants showed a significant reduction in FRET upon Pi treatment. Under the same experimental conditions, however, the cp-FLIPPi-Null plants did not show a significant change in FRET ratios at the different Pi concentrations, indicating negligible response to Pi treatment (Figure 2d). By normalizing the FRET ratios of both variants to P_0_, the cpFLIPPi5.3m sensor exhibited a significant reduction in FRET values (of ~49%), of which the non-specific variation accounts for only ~7% of variation. Together, these observations indicated that the cpFLIPPi sensor plants gave Pi-dependent FRET responses, thus supporting functional Pi-sensing capability in the cpFLIPPi5.3m *Arabidopsis* plants. These findings also underscore the specificity and sensitivity of the cpFLIPPi5.3m sensor to Pi, at least in the range of Pi concentrations assayed in this work.

Additionally, time-course live imaging was carried out to investigate real-time cytosolic Pi fluctuations in the roots of *Arabidopsis* plants expressing the cpFLIPPi5.3m sensor. Two-week-old seedlings conditioned in low Pi (0.025 mM Pi) were supplied with high Pi (2.5 mM Pi) on the slide setup for live imaging and measured over 120 min. Pi-induced changes in cytosolic Pi levels were monitored by recording FRET ratios (cpVenus/eCFP) every 15 min. As shown in Figure 2e, cpFLIPPi5.3m plants displayed a statistically significant decrease in FRET ratios during the first 60 min, suggesting a rapid Pi accumulation in the cytosol. This initial response was followed by a plateau phase until 120 min, where FRET ratios remained relatively unchanged, suggesting a stabilization of cytosolic Pi levels (Figure 2e). Based on these observations, the initial 60 min window was selected for subsequent real-time imaging of Pi responses in *Arabidopsis* root epidermal cells.

### 2.3. Pi Treatment Increases Cytosolic Pi Levels in Rice Roots Reflected by Decreased FRET Emission

To investigate the effect of Pi availability on cytosolic Pi concentration in epidermal cells of rice roots, plants were grown in half-strength MS medium for the first 7 days and were subsequently exposed to increasing concentrations of Pi (e.g., P_0_, P_0.025_, P_0.25_, and P_2.5_) for the next 3 days. Pi measurements using the spectrophotometric method confirmed increased Pi content in the root system when increasing Pi supply to the rice plants (Figure 3a), thus confirming that plants respond to Pi treatment distinctively. Rice seedlings were then analyzed for FRET emissions in root epidermal cells in the transition–elongation zone using confocal microscopy. As previously observed in cpFLIPPi5.3m *Arabidopsis* plants, a progressive decrease in FRET ratios (cpVenus/eCFP ratios) was observed with increasing Pi concentrations, indicating an increase in cytosolic Pi levels (Figure 3b).

To further verify that the observed changes reflect a response to external Pi treatment, FRET ratios were measured in rice seedlings grown for 7 days (in half-strength MS medium) and after 3 days under defined Pi regimes (P_0.025_, P_0.25_, and P_2.5_). Seedlings exposed to lower Pi concentrations (e.g., P_0.025_) displayed elevated FRET ratios, whereas those supplied with higher Pi concentrations (e.g., P_2.5_) showed moderately reduced ratios (Figure 3c). As previously verified in *Arabidopsis*, the sensor’s specificity to Pi of the cpFLIPPi5.3m lines was evaluated by comparing their FRET values to those in the Pi-insensitive cpFLIPPi-Null variant lines. In cpFLIPPi-Null seedlings, FRET ratios remained relatively unchanged across Pi treatments (P_0_ and P_2.5_), whereas cpFLIPPi5.3m seedlings exhibited a significant reduction in FRET values (Figure 3d). In this way, the FRET values normalized to P_0_ revealed a reduction of ~48% in cpFLIPPi5.3m plants, of which ~44% is Pi-specific response and ~4% is non-specific response. Again, these results confirmed the sensor’s high specificity to Pi in rice.

Extending this, a time-course live imaging was performed to observe the FRET ratios dynamically, in response to Pi application in rice roots. 10-day-old cpFLIPPi5.3m rice seedlings conditioned in 0.025 mM Pi for 3 days were applied with a solution of 2.5 mM Pi on the slide setup. These roots were monitored every 15 min over a period of 120 min for Pi-induced changes in FRET ratios (cpVenus/eCFP). The average FRET ratio decreased significantly over the first 60 min and, later on, stabilized by 120 min (Figure 3e). As noted in *Arabidopsis*, the time frame of 60 min was also followed for further time-course experiments in rice.

Collectively, these results demonstrated that the cpFLIPPi5.3m sensor performs robustly in rice plants, recapitulating the Pi-dependent FRET responses previously validated in *Arabidopsis*. The observed Pi-specific change of ~44% in rice and ~42% in *Arabidopsis* underscore the reliability and responsiveness of the FLIPPi5.3m biosensor in both monocot and dicot plant systems.

### 2.4. Cytosolic Pi Levels Increase in Response to Treatment with Defense-Related Hormones and Elicitors of Immune Responses in Arabidopsis and Rice Roots

In previous studies, we reported that Pi supply has an effect on disease resistance in both *Arabidopsis* and rice plants [9,10]. In this work, we investigated the feasibility of using cpFLIPPi5.3m sensor plants to monitor alterations in Pi content in response to treatment with key plant hormones involved in plant immunity and elicitors of plant immune responses. To this end, sensor plants were treated with the defense-related hormones salicylic acid (SA), methyl-jasmonate (MeJA), and 1-aminocyclopropane 1-carboxylic acid (ACC, the direct precursor of the plant hormone ethylene). FRET ratios were monitored over 60 min of exposure to treatment solutions. Parallelly, the treated plants were analyzed for Pi content using the spectrophotometric method.

**Figure 4:**
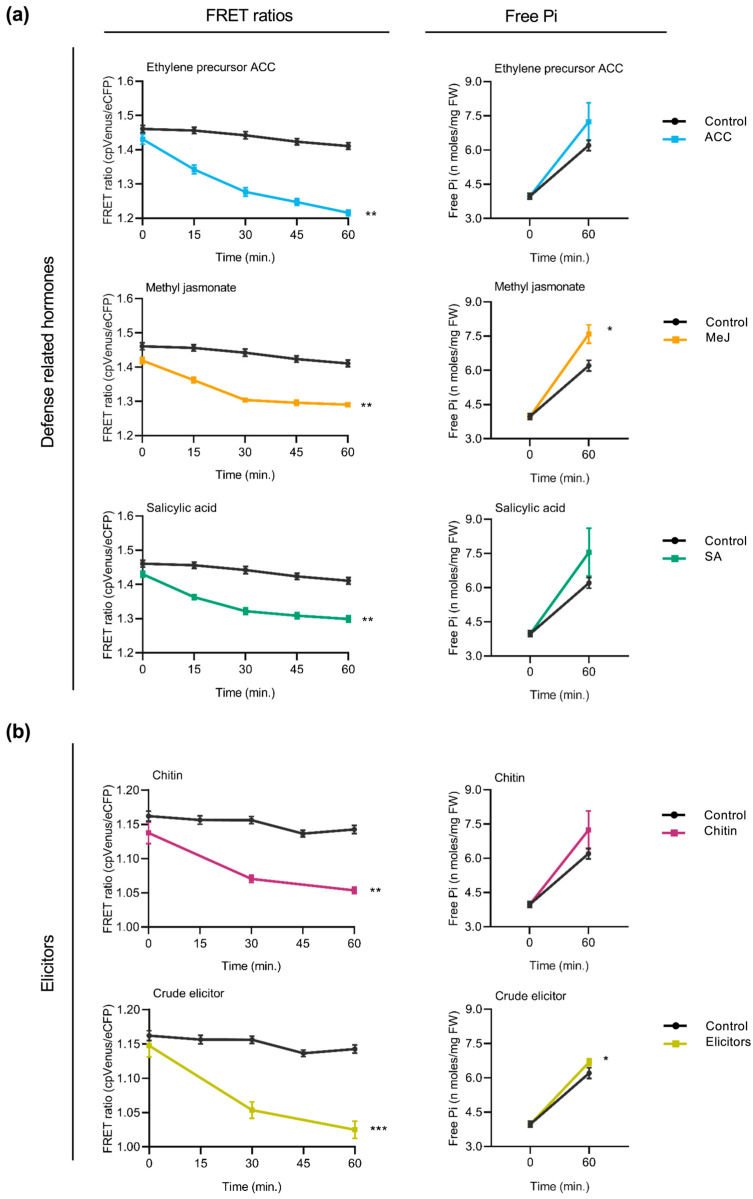
Effect of treatment with defense-related hormones and elicitors of immune responses on cytosolic Pi concentrations in *Arabidopsis*. FRET ratios (cpVenus/eCFP) were measured in the root epidermal cells over 60 min of treatment. Two-week-old FLIPPi5.3m *Arabidopsis* plants conditioned in a 0.25 mM Pi regime were treated with the compound of interest. Control seedlings were treated with 0.25 mM Pi. Three biological replicates were examined, each one consisting of at least six seedlings for FRET imaging, and twelve seedlings were pooled for spectrophotometric quantification of Pi. Error bars represent ±SEM. Statistical significance was tested by Student’s *t*-test with * *p* ≤ 0.05, ** *p* ≤ 0.01, and *** *p* ≤ 0.001. The controls are in black color and the respective treatments are in colour as per the graph. Details of the growth conditions and treatments are summarized in Table S1. (**a**) Treatment with defense-related hormones. Left panel, FRET ratios in the roots of FLIPPi5.3m *Arabidopsis* subjected to treatment with SA, MeJA, or the ethylene precursor (ACC) (10 µM in 0.25 mM Pi) compared to control plants (0.25 mM Pi). Right panel, free Pi content of the corresponding treated roots at 0 min and 60 min. (**b**) Treatment with elicitors of immune responses. Left panel, FRET ratios in the roots of FLIPPi5.3m *Arabidopsis* subjected to treatment with either chitin (100 µg/mL in 0.25 mM Pi) or crude elicitors obtained from the *Arabidopsis* root pathogen *Fusarium oxysporum* f. sp. *conglutinans* (100 µg/mL in 0.25 mM Pi). Right panel, free Pi content of the corresponding treated roots at 0 min and 60 min.

In *Arabidopsis* cpFLIPPi5.3 plants, treatment with defense-related hormones decreased FRET ratios (cpVenus/eCFP) in root epidermal cells, suggesting an increase in cytosolic Pi levels (Figure 4a, left panels). The decrease in FRET ratios was observed consistently across all three hormone treatments. Increased cytosolic Pi levels observed by live imaging of Pi levels correlated well with an increase in the Pi content of hormone-treated *Arabidopsis* roots (Figure 4a, right panels).

Two types of elicitors of immune responses were used to explore alterations in cytosolic Pi levels in the roots of *Arabidopsis* plants. On one hand, the cpFLIPPi5.3m-expressing *Arabidopsis* plants were treated with chitin, a component of fungal cell walls. Chitin is also a well-characterized pathogen-associated molecular patterns (PAMPs) molecule that triggers pattern-triggered immunity (PTI) responses in plants. On the other hand, the cpFLIPPi5.3m *Arabidopsis* plants were treated with crude extracts obtained from the root-infecting fungus *Fusarium oxysporum* f. sp. *conglutinans*. *Fusarium* species are known pathogens infecting more than a hundred plant species, including members of the Brassicaceae family [28]. Both chitin and fungal elicitors were found to provoke a decrease in FRET ratios compared to the control plants (Figure 4b, left panels), indicating an increase in cytosolic free Pi. These observations were independently validated by biochemical quantification of free Pi levels (Figure 4b, right panels). Consistently, the treatment of *Arabidopsis* plants with either chitin or fungal elicitors led to an increase in Pi levels in the root epidermal cells of *Arabidopsis*.

Together, these results suggest that treatment of *Arabidopsis* plants with the defense hormones (SA, MeJA, and the ethylene precursor ACC) or elicitors that trigger immune responses in plants is accompanied by an increase in cytosolic Pi levels. Furthermore, these findings further support crosstalk between Pi signaling and immune signaling in *Arabidopsis* plants.

**Figure 5:**
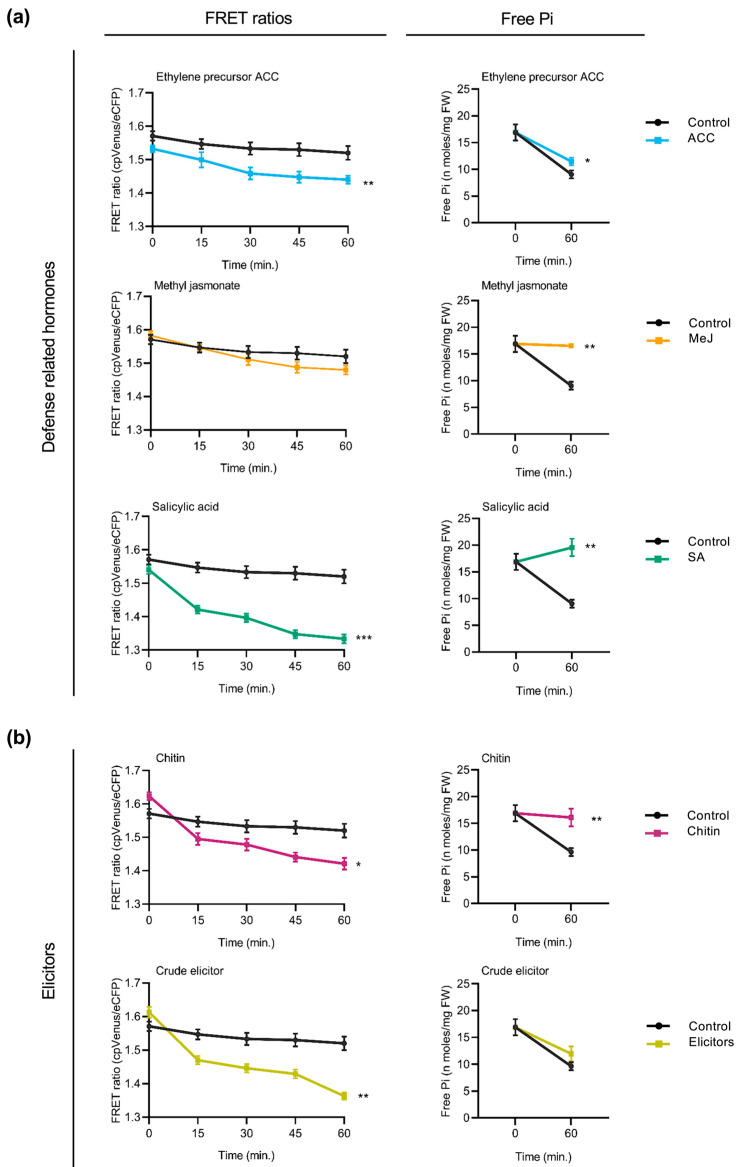
Influence of defense-related phytohormones and fungal elicitors on cytosolic Pi content reflected by FRET ratios and free Pi content in the roots of FLIPPi5.3m rice seedlings. Ten-day-old FLIPPi5.3m sensor rice seedlings grown under a Pi 0.25 mM regime were treated with SA, MeJA, or the ethylene precursor (ACC). Control seedlings were treated with 0.25 mM Pi. FRET ratios (cpVenus/eCFP) were recorded every 15 min over a period of 60 min. Three biological replicates were examined, each one consisting of at least six seedlings for FRET imaging, and twelve seedlings pooled for spectrophotometric quantification of Pi. Error bars represent ±SEM. Statistical significance was tested by Student’s *t*-test with * *p* ≤ 0.05, ** *p* ≤ 0.01, and *** *p* ≤ 0.001. The controls are in black color and the respective treatments are in colour as per the graph. Details of growth conditions and treatments are summarized in Table S2. (**a**) Effect of treatment with the defense-related phytohormones. Left panels show FRET ratios of treatment with either SA, MeJA, or the ethylene precursor ACC (10 µM each) compared to the control plants. Right panels show corresponding measurements of free Pi content at 0 min and 60 min after treatment. (**b**) Effect of treatment with elicitors of immune responses. Left panels show FRET ratios of rice seedlings in response to treatment with either chitin (100 µg/mL in 0.25 mM Pi) or with crude elicitors of rice root pathogen *Fusarium fujikuroi* (100 µg/mL in 0.25 mM Pi) compared to the controls (0.25 mM Pi). Right panels show corresponding measurements of free Pi content using the spectrophotometric method at 0 min and 60 min after treatment.

Next, we examined whether treatment with defense-related hormones influences cytosolic Pi content in rice roots. FRET measurements taken at regular intervals revealed a progressive decrease in FRET ratios (cpVenus/eCFP) in response to each hormone treatment compared to the control group (Figure 5a, left panels), suggesting an increase in cytosolic Pi levels. The extent of FRET ratio reduction varied slightly between hormones, with the most pronounced effect observed upon SA treatment. Consistent with the observed reduction in FRET ratios, spectrophotometric determinations of Pi content revealed an increase in Pi in the hormone-treated plants relative to the untreated controls (Figure 5a, right panels).

The FRET response to elicitors of plant immune responses was also examined in rice plants. These included treatment with chitin or treatment with crude elicitors from a rice root fungal pathogen *Fusarium fujikuroi*. Compared to control plants, cpFLIPPi5.3m rice seedlings that had been treated with either chitin or fungal elicitors showed a progressive decrease in FRET ratios (Figure 5a, left panels), indicative of increased cytosolic Pi levels. Parallelly, free Pi content measurements of the root tissues confirmed an increase in Pi content in both chitin- and *F. fujikuroi* elicitor-treated plants relative to the controls (Figure 5b, right panels).

Altogether, the above findings highlight that treatment with hormones that orchestrate defense responses as well as treatment with compounds triggering immune responses (e.g., chitin and fungal elicitors) provoke an increase in cytosolic phosphate levels in rice roots. Notably, these patterns are consistent with the changes observed in *Arabidopsis*, suggesting a conserved mechanism underlying the integration of Pi signaling and immune responses across the two plant species, a monocot and a dicot species.

### 2.5. Phosphite Application Alters Cytosolic Pi Dynamics in Roots of Arabidopsis and Rice Plants

**Figure 6:**
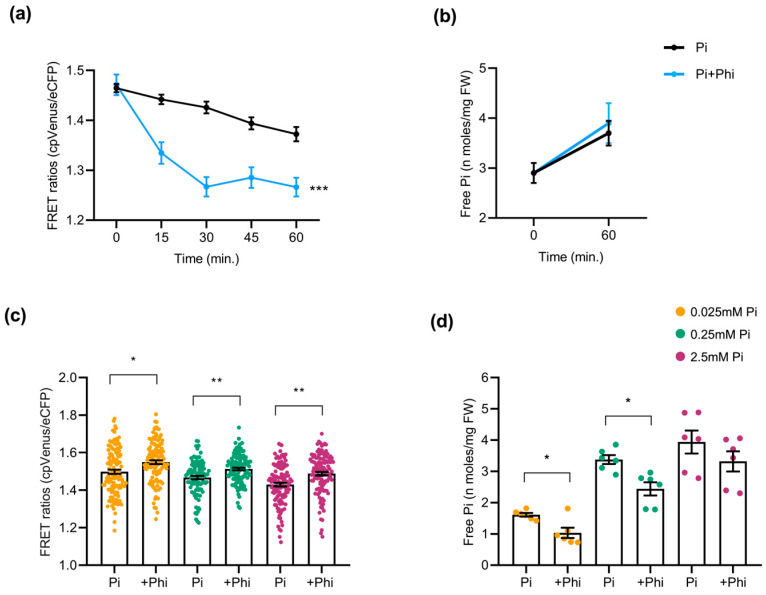
Effect of phosphite (Phi) application on Pi content in *Arabidopsis* roots. At least 10 plants were used per condition for FRET analysis, and 24 seedlings were pooled for free Pi measurement of *Arabidopsis* roots. Bars represent ± SEM. Statistical significance was tested by Student’s *t*-test with * *p* ≤ 0.05, ** *p* ≤ 0.01, and *** *p* ≤ 0.001. Phi treatment conditions of *Arabidopsis* are detailed in Table S1. (**a**) FRET ratios of cpFLIPPi5.3m *Arabidopsis* roots exposed to a combination of Pi and Phi (0.025 mM Pi + 0.0025 mM Phi) compared to Pi-only (0.025 mM Pi) as control, monitored over 60 min. (**b**) Corresponding free Pi content measured by spectrophotometry at 0 min and 60 min post treatment. (**c**) FRET ratios of cpFLIPPi5.3m *Arabidopsis* roots conditioned in Pi alone (P_0.025_, P_0.25_, and P_2.5_) or their counterparts in the presence of Phi supplementation (at 1/10th ratio relative to the corresponding Pi concentration). (**d**) Free Pi content of cpFLIPPi5.3m *Arabidopsis* roots from the same batch of plants measured by spectrophotometry.

Phi, an analog of Pi, is taken up by plants via phosphate transporters through roots, similarly to Pi, but cannot be metabolized. Owing to its structural similarity with Pi, we hypothesized that Phi application might provoke alterations in the Pi content of roots. Accordingly, in this work, we monitored cytosolic Pi using the FLIPPi-expressing *Arabidopsis* and rice plants exposed to Phi under different conditions. For this, 2-week-old cpFLIPPi5.3m *Arabidopsis* seedlings conditioned in low Pi (0.025 mM) were treated with either Pi-only (0.025 mM Pi) as controls, or with a combination of Pi and Phi (0.025 mM Pi + 0.0025 mM Phi) as Phi treatment (detailed in Table S1). Time-course live imaging over 60 min revealed a rapid decline in FRET ratios in Phi-exposed roots compared with the Pi-only controls, consistent with a transient increase in cytosolic Pi (Figure 6a). This observation was supported by the free Pi content of roots measured spectrophotometrically at 0 min and 60 min post treatment, although the resolution of biochemical quantification was not as precise as in FRET (Figure 6b). Given the structural similarity between Pi and Phi, we cannot rule out the potential interaction of Phi with the FLIPPi sensor. The FRET trends observed here corelate with spectrophotometric free Pi measurements, indicating that the alterations more likely reflect changes in cytosolic Pi.

To test whether the changes triggered by Phi are dependent on Pi availability, cpFLIPPi *Arabidopsis* plants initially grown for one week in half-strength MS medium were later transferred to medium containing either Pi alone (P_0_, P_0.025_, P_0.25_, or P_2.5_), or a combination of the corresponding Pi concentrations plus Phi (1/10th molar ratio relative to Pi in all the conditions) for another week (detailed in Table S1). Under these conditions, FRET ratios of Phi-supplemented *Arabidopsis* roots were higher compared to their Pi-only counterpart controls, suggesting reduced cytosolic Pi levels (Figure 6c). Direct spectrophotometric measurements of the Pi content supported the sensor data, as the roots of Phi-treated plants accumulated significantly less Pi than those supplied with Pi alone (Figure 6d).

**Figure 7:**
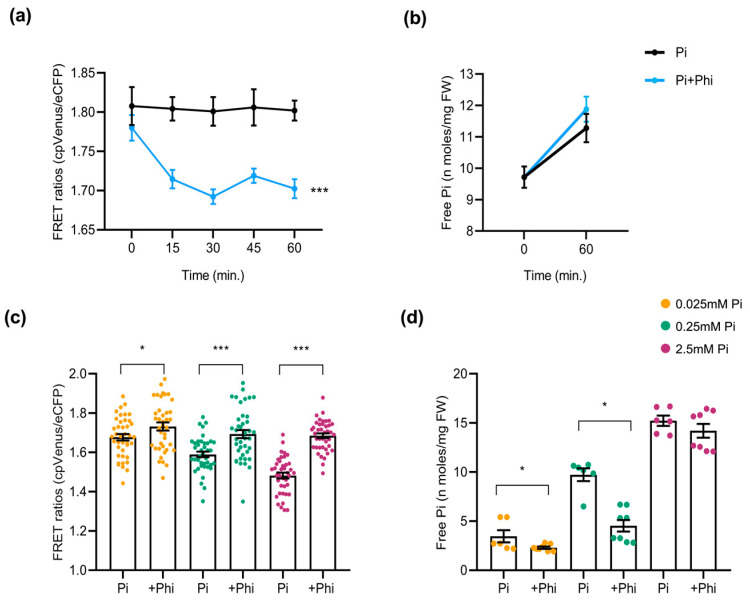
Effect of Phi application on the cytosolic Pi content in rice roots. At least 6 plants were used per condition for FRET analysis, and 12 seedlings for free Pi measurement of rice roots. The error bars represent the ± SEM. Statistical significance was tested by Student’s *t*-test * *p* ≤ 0.05 and *** *p* ≤ 0.001. Phi treatment conditions of rice are summarized in Table S2. (**a**) FRET ratios of cpFLIPPi5.3m rice plants conditioned in 0.025 mM Pi for 3 days and exposed to Phi treatment solution (0.025 mM Pi + 0.0025 mM Phi) compared to the control Pi solution (0.025 mM Pi), monitored over 60 min. (**b**) Corresponding free Pi content measured spectrophotometrically at 0 min and 60 min post treatment. (**c**) FRET ratios of cpFLIPPi5.3m rice roots grown under Pi alone (0.025, 0.25, or 2.5 mM) or their counterparts supplemented with Phi (1/10th molar ratio relative Pi concentration). (**d**) Corresponding free Pi content of cpFLIPPi5.3m rice roots measured spectrophotometrically.

As carried out in *Arabidopsis* sensor plants, we examined the effect of Phi on Pi content in rice plants. For this, 10-day-old cpFLIPPi5.3m rice seedlings that were grown in low Pi (0.025 mM) were exposed to a Phi treatment solution (0.025 mM Pi + 0.0025 mM Phi) or control solution (0.025 mM Pi). The FRET ratios of the Phi-exposed seedlings showed a rapid decline over 60 min, indicating an acute rise in cytosolic Pi compared to the Pi-only controls (Figure 7a). This observation was compared parallelly to the free Pi content measured at 0 min and 60 min after treatment with Phi solution, implying an increase in cytosolic Pi (Figure 7b). When rice plants were grown under different Pi regimes (0.025, 0.25, and 2.5 mM) with or without Phi supplementation (1/10th molar ratio), the long-term response resembled that observed in *Arabidopsis*. Phi addition increased FRET ratios, consistent with lower cytosolic Pi (Figure 7c). This upward shift in FRET ratios suggests a relative decrease in cytosolic Pi. The quantification of free Pi content in roots mirrored FRET results, as Pi+Phi-treated seedlings exhibited significantly lower Pi accumulation than those treated with Pi alone (Figure 7d).

Taken together, our results indicate that Phi application has an effect on cytosolic Pi levels in root epidermal cells of both *Arabidopsis* and rice roots, in a time- and context-dependent manner. These findings suggest that Phi might interfere with Pi transport and accumulation. Furthermore, the possibility that Phi binds to the PiBP of the FLIPPi sensor should be considered, an aspect that remains to be investigated. Additional studies using Phi-insensitive control sensors and molecular analyses would be required to conclusively determine the mechanism of Phi on Pi homeostasis. In summary, the results obtained in this work underscore the sensitivity, specificity, and practical applicability of the FLIPPi5.3m biosensor for quantitative real-time visualization of Pi in roots of *Arabidopsis* and rice plants.

## 3. Discussion

P is a necessary macronutrient for plant growth and development. Although the overall content of P in soils is generally high, its low bioavailability limits crop yield in many agricultural ecosystems. As a consequence, Pi fertilizers are routinely used to support high crop yields, which also cause serious environmental problems. Both Pi excess and deficiency might cause nutritional imbalances that negatively impact plant growth and productivity, as well as adaptation to environmental stress. Maintaining proper Pi levels in plants ensures healthy growth and high yields in crops. Along with this, the use of tools to follow Pi homeostasis in crop plants is a necessity in crop research and will provide solutions for the development of a more sustainable use of Pi fertilizers in agriculture. In this study, we provide evidence on the effectiveness of FRET-based phosphate biosensors for real-time visualization of cytosolic Pi content in the roots of *Arabidopsis* and rice plants. The use of a FLIPPi biosensor provided robust insights into Pi dynamics in response to Pi availability as well as in response to molecules involved in the regulation of plant immune responses. Equally, the FLIPPi biosensor has proven to be useful in determining the impact of Phi application on cytosolic Pi levels in the roots of *Arabidopsis* and rice plants.

Live imaging of roots revealed a significant reduction in FRET ratios with increasing Pi availability in both plant species, aligning with the anticipated sensor behavior, where Pi binding induces a conformational change, reducing energy transfer between eCFP and cpVenus [26,27]. The specificity and reliability of the cpFLIPPi sensor for Pi was validated by comparing the Pi-sensitive cpFLIPPi5.3m sensor against the Pi-insensitive cpFLIPPi-null variant and further confirmed by spectrophotometric quantification of the Pi content in roots. While the null variant exhibited minimal FRET ratio changes irrespective of Pi concentration, the cpFLIPPi5.3m variant consistently showed significant, concentration-dependent Pi changes. Deviations between FRET-based cytosolic Pi measurements and spectrophotometric determinations of free Pi could be noted in some cases, such as in Figure 5. This incongruity likely reflects the different sensitivities and spatial resolutions of the two methods (FRET imaging captures real-time cytosolic Pi changes at the cellular level, whereas spectrophotometry provides a bulk tissue estimate of soluble Pi). While both approaches capture the overall trends in Pi dynamics, a precise correlation between them is not presumable. The cpFLIPPi5.3m sensor has previously been reported to be reversible and sensitive across physiological pH ranges, supporting the reliability of the FRET-based trends observed here, while also considering the minimal impact of non-specific changes [23,26]. Additionally, time-course studies on Pi-treated *Arabidopsis* plants revealed a continuous decrease in FRET ratios, by about 20% in *Arabidopsis* roots and by about 30% in rice roots, after the first hour of Pi treatment, indicative of cytosolic Pi accumulation. This response was followed by a plateau phase suggestive of homeostatic adjustments either by internal Pi redistribution processes (e.g., vacuole–cytosol redistribution), or through the modulation of Pi uptake mechanisms (e.g., the activity of Pi transporters). Interestingly, the FRET responses of *Arabidopsis* and rice roots to Pi treatment exhibited similar trends. Collectively, the results presented here demonstrated that FLIPPi sensors can reliably monitor in vivo cytosolic Pi fluctuations in root cells, providing an indispensable tool for detailed studies on Pi homeostasis in *Arabidopsis* and rice, model plants for studies in dicotyledonous and monocotyledonous species. FLIPPi biosensors also pose a promising tool for studies on Pi metabolism and nutrition in other crop species, e.g., in cereal crops. Clearly, a better understanding of Pi dynamics will provide essential insights for crop breeding strategies aimed at enhancing phosphate-use efficiency and stress resilience.

Notably, in this study we demonstrated that Pi nutrition and immunity are intricately linked. Treatment with defense-related hormones and elicitors of immune responses provoked an increase in cytosolic Pi levels in both *Arabidopsis* and rice plants. This rise in Pi likely reflects an enhanced uptake or mobilization of internal Pi pools to sustain the high energy demand associated with defense activation. Defense responses, including the biosynthesis of antimicrobial metabolites, reinforcement of cell walls, and activation of phosphorylation cascades require substantial ATP and phosphorylated intermediates, which can depend immediately on cellular Pi availability [29]. Pi availability plays a pivotal role in shaping plant immune responses, acting both as a metabolic signal and as a regulator of hormone-mediated defense pathways. Conversely, Pi nutrition also modulates the production and signaling of these same defense-related hormones [30]. Elevated Pi availability has been shown to enhance SA and JA accumulation in *Arabidopsis* [10], thereby promoting resistance to necrotrophic and hemibiotrophic pathogens such as *Plectosphaerella cucumerina* and *Colletotrichum higginsianum*. In contrast, in rice, elevated Pi levels often increase susceptibility to fungal infection by *Magnaporthe oryzae* and *Fusarium fujikuroi* [9,11]. This contrasting response likely reflects species-specific regulatory networks in Pi–hormone crosstalk: while *Arabidopsis* tends to activate defense hormone pathways under high Pi, rice appears to prioritize growth, leading to a partial suppression of immune signaling and increased pathogen susceptibility. Reinforcing this notion, in this study, treatment with inducers of plant immunity (e.g., fungal elicitors and chitin) triggers an increase in cytosolic Pi in *Arabidopsis* and rice. In line with this, pathogen recognition, specifically PAMPs recognition, by plant receptors (PRRs) is known to provoke Pi influx into the plant cell, leading to downstream signaling cascades in which diverse phosphorylation processes participate. Pi fluxes are then closely integrated with immune responses, potentially modulating disease resistance in plants. Together, this piece of information suggests that both hormone signaling and elicitor-induced signaling pathways intersect with Pi signaling, reflecting complex nutrient–immunity crosstalk in plants, both monocots and dicots. The biological significance of these observations lies in the plant’s trade-offs between nutrient status, energy metabolism, and defense prioritization. This illustrates the complex and intricate interplay between the mechanisms underlying Pi homeostasis and immunity in plants, while emphasizing the need to further investigate mechanisms underlying Pi nutrition and disease resistance in plants. It would therefore be of interest to extend the use of FLIPPi sensors to other crop species.

Moreover, the results presented here on the effect of Phi application on Pi levels offered further insights into the complexity of Pi homeostasis in *Arabidopsis* and rice plants with potential implications for agriculture. Real-time imaging experiments revealed that Phi exposure triggered a reduction in FRET ratios, suggesting a spike of cytosolic Pi. This could refer to a response of Pi redistribution or enhanced Pi uptake, suggesting nuanced regulatory mechanisms possibly activated to counterbalance Phi-induced disruptions. Further, treatment with a combination of Pi + Phi (1/10th molar ratio) resulted in an increase in FRET ratios, indicative of reduced cytosolic Pi levels relative to plants that had been treated with Pi alone, supported by spectrophotometric quantification of Pi content. This response was observed both in *Arabidopsis* and rice roots. Different possibilities can be reasoned to explain these observations. Firstly, Phi might interfere with Pi binding to the FLIPPi sensor or bind by itself, thus influencing sensor behavior. The Pi-binding domain of the FLIPPi sensor might not bind to Phi directly with the same affinity, yet cellular changes triggered by Phi might affect Pi levels in a way that is detectable by the sensor. Future studies are required to determine the potential sensitivity or affinity of FLIPPi sensors for Phi compared to Pi. Secondly, Phi might disrupt the function of Pi transporters, leading to altered Pi uptake and/or distribution contributing to reduced Pi levels in the roots of Pi+Phi-treated plants. Alternatively, given that Phi is not metabolically usable by most plants, its presence might potentially disrupt processes involved in the maintenance of Pi homeostasis, a phenomenon that deserves further investigation. Additionally, increasing evidence exists for the potential of Phi to potentiate natural plant defenses against a range of diseases, with this compound acting as an inducer of resistance via defense priming [31]. The beneficial effects of Phi in *A. thaliana* were reported to be mediated by the activation of hormone signaling pathways (e.g., SA, JA, and Abscisic acid signaling) [31].

In conclusion, this study demonstrates the usefulness of FLIPPi biosensors to elucidate Pi dynamics in vivo, substantially advancing our understanding of the integration of Pi signaling pathways and immune responses. The successful implementation of FLIPPi5.3m biosensor technology across two evolutionarily distant plant species, *Arabidopsis* and rice, highlights its broad applicability for Pi signaling studies in plants. From the practical point of view, the integration of FLIPPi sensors into rice research opens exciting possibilities to monitor and track Pi nutrition, ultimately facilitating the development of crops with improved nutrient-use efficiency and stress tolerance. The insights gained from this research also provide a robust foundation for rationally optimizing fertilizer use in rice cultivation for the development of sustainable production systems.

## 4. Materials and Methods

### 4.1. Plant Material and Growth Conditions

*Arabidopsis thaliana* (ecotype Columbia-0, Col-0) background plants were grown at 22 °C ± 2 °C with an 8 h photoperiod (110 µmol m^−2^ s^−1^ photon flux density) and 60% relative humidity. *Arabidopsis* seeds were surface-sterilized with a solution of 1% sodium hypochlorite plus 0.05% SDS and extensively rinsed with sterile water. *Arabidopsis* seeds underwent cold stratification at 4 °C for 3–4 days to synchronize germination. Rice (*Oryza sativa*, cv. Tainung 67) background plants were grown at 28 °C ± 2 °C under a 12 h photoperiod (150 µmol m^−2^ s^−1^ photon flux density) and 60% relative humidity. For this, rice seeds were surface-sterilized in 70% ethanol (1 min), washed briefly, and then agitated in 4% sodium hypochlorite containing 0.5% TWEEN^®^ 20 (Calbiochem^®^, Merck KGaA, Darmstadt, Germany) for 30 min, followed by washing with sterile water. Rice seeds were incubated at 37 °C in darkness for 3–4 days to ensure even germination. Measurements of root length were carried out by image analysis using the ImageJ/Fiji software version 2.16.0/1.54p (http://fiji.sc/Fiji).

### 4.2. Generation of Transgenic Arabidopsis and Rice Plants Expressing FLIPPi Sensors

Transgenic *Arabidopsis* and rice lines constitutively expressing the cpFLIPPi5.3m sensor were used to investigate cytosolic Pi changes in response to external treatments. *Arabidopsis* plants expressing the Pi-insensitive variant, cpFLIPPi-Null, were included as negative controls [26]. Plasmids containing either the cpFLIPPi5.3m sensor or the FLIPPi-Null variant (pCN_cpFLIPPi5.3 and pCN_FLIPPi-Null, respectively), as well as transgenic *Arabidopsis* lines constitutively expressing one or another gene, were kindly provided by Dr. Wayne K. Versaw (Department of Biology, Texas A&M University, USA) [23]. The FLIPPi sensors consist of a cyanobacterial Pi-binding protein (PiBP) fused to an enhanced cyan fluorescent protein (eCFP) and a circularly permuted (cp) yellow fluorescent protein Venus (cpVenus) (eCFP::PiBP::cpVenus). The cpFLIPPi-Null variant contained mutations in a 6-amino-acid stretch (positions 18–23) within a 43-amino-acid conserved region, which abolished Pi-dependent conformational changes, hence FRET ratios in this mutant remained unchanged upon Pi addition [26]. For constitutive expression of FLIPPi genes in *Arabidopsis*, the *Ubiquitin 10* (At*Ubi10*) promoter was used [23]. The *Arabidopsis* FLIPPi lines used in this study were previously described [23]. Homozygous T3 *Arabidopsis* lines were verified and used for further FRET analysis (cpFLIPPi 5.3m line 3.1 and line 8.3; cpFLIPPi-Null line 5.1).

Rice sensor lines harboring either the cpFLIPPi5.m or the cpFLIPPi-null sensor genes were constitutively expressed in rice under the control of the maize *Ubiquitin 1* (*Ubi1*) promoter. For this, the coding sequences of cpFLIPPi genes were PCR-amplified from pCN_cpFLIPPi5.3m and pCN_cpFLIPPi-Null plasmids and cloned into a modified pCAMBIA1300 binary vector containing maize *Ubiquitin1* (*Ubi1*) promoter and *nopaline synthase* (*NOS*) terminator and *hptII* (*hygromycin phosphotransferase*) as the selection marker in the T-DNA. Primers used for PCR and cloning are listed in Table S3. For rice transformation, the generated plasmid constructs harboring FLIPPi sensors were transferred to the *Agrobacterium tumefaciens* EAH105 strain. Transgenic rice lines were produced by *Agrobacterium*-mediated transformation of embryogenic calli derived from mature embryos. The stability of transgene expression was monitored through successive generations by RT-qPCR. Primers used for the expression analysis of PiBP are listed in Table S3. Independently generated transgenic lines (T3 homozygous lines, cpFLIPPi5.3m line 10; and line 15, and cpFLIPPi-Null line 6 and line 8) were then utilized for live imaging of cytosolic Pi.

Phenotypically, *Arabidopsis* cpFLIPPi5.3m, cpFLIPPi-Null, and wild-type plants, as well as rice cpFLIPPi5.3m, cpFLIPPi-Null, and wild-type plants, were indistinguishable, indicating that sensor expression did not interfere with normal development.

### 4.3. RT-qPCR

Total RNA was extracted using the Maxwell RSC Plant RNA kit (Promega, Madison, WI, USA). First-strand cDNA was synthesized from DNase-treated total RNA (1 µg) with the High Capacity cDNA reverse transcription kit (Applied Biosystems, Walthman, MA, USA). RT-qPCRs were carried out in optical 96-well plates using SYBR^®^ green in a Light Cycler 480 (Roche, Basel, Switzerland). PCR primers were designed with the Primer-BLAST tool (https://www.ncbi.nlm.nih.gov/tools/primer-blast/). The rice *Ubiquitin 1* gene (Os06g0681400) was used for normalization of transcript levels. The ΔCt method was used to calculate relative expression levels. Primers used for RT-qPCR are shown in Table S3. Two independent experiments were conducted, each one consisting of at least 4 biological replicates (pool of 6 plants for each replicate).

### 4.4. Pi Treatments

Seedlings were initially grown for 1 week on half-strength MS medium (Duchefa Biochemie, Haarlem, Netherlands). For phosphate (Pi) treatments, seedlings were transferred to modified half-strength Hoagland’s medium with different Pi concentrations for 1 week (*Arabidopsis*) or 3 days (rice) before analysis. The Pi concentrations used were 0 mM Pi (no Pi added to the medium), 0.025 mM Pi, 0.25 mM Pi, and 2.5 mM Pi. Pi concentrations used for time-course experiments are summarized in Table S1 and Table S2 for *Arabidopsis* and rice, respectively.

### 4.5. Phosphate Quantification

Free inorganic phosphate (Pi) content of either *Arabidopsis* or rice roots were quantified using a colorimetric assay based on the formation of a molybdenum blue complex [32,33]. Pooled tissues (10 mg; 10 plants per pool) were flash-frozen in liquid nitrogen and homogenized with a TissueLyser II (QIAGEN, Hilden, Germany). Samples were treated and incubated in 1 mL of 1% glacial acetic acid. For Pi quantification, 0.3 mL of the extract was mixed with 0.7 mL of a reagent composed of 10% ascorbic acid and 0.42% ammonium molybdate (1:6 ratio) in 1N H_2_SO_4_. The intensity of the blue complex formed with free PO_4_^3−^ was measured at 820 nm using the spectrophotometer SpectraMax^®^ (Molecular Devices LLC, Sunnyvale, CA, USA)plate reader. Pi concentrations were calculated using a standard curve generated from known phosphate standards.

### 4.6. Hormone and Elicitor Treatments

Stock solutions of the plant hormones salicylic acid (SA) and methyl jasmonate (MeJA) were prepared in absolute ethanol (at a concentration of 100 mM). The stock solution of the ethylene precursor 1-aminocyclopropane-1-carboxylic acid (ACC) was prepared in sterile water. All stocks were filter-sterilized with 0.2 μm Corning^®^ syringe filters and stored at −20 °C. Working solutions (at 10 µM concentration) were freshly prepared by diluting these stocks into half-strength Hoagland’s medium, pH 5.8, containing 0.1% (*v*/*v*) agar, and used for imaging of sensor plants.

Two different types of elicitors were used in this study, namely, chitin and crude elicitors prepared from a fungal pathogen. Chitin, a major component of fungal cell walls, serves as a PAMP for the activation of immune responses in plants. Chitin was prepared by dissolving commercial shrimp chitin (1 g) in 1% acetic acid and heating for a few minutes to dissolve. The volume was made up to 10 mL using milli-Q H_2_O, and this solution was later used to prepare the working solution (100 µg/mL) for chitin treatment (in half-strength Hoagland’s medium, pH 5.8, containing 0.1% (*v*/*v*) agar).

Crude elicitors were prepared from the *Arabidopsis* root pathogen *Fusarium oxysporum* f. sp. *conglutinans* and the rice root pathogen *Fusarium fujikuroi* by standard procedures. Briefly, the fungus was grown in liquid medium (PDA) for 2–3 weeks at 28 °C. Mycelium was collected from liquid cultures with filter cloth, washed with H_2_O, and frozen until use (−20 °C). The fungal mycelium was ultrasonicated (amplitude 40–50%, 5–10 s pulse, with a total cycle of 10–15 min). The samples were autoclaved (120 °C for 20 min at 1 atm). To obtain a crude extract, the samples were lyophilized and stored at −20 °C until use. Working solutions of 100 µg/mL concentrations were freshly prepared using freeze-dried fungal mycelium in half-strength Hoagland’s medium, pH 5.8, containing 0.1% agar. Hormone and elicitor treatment conditions are summarized in Table S2 and Table S3 for *Arabidopsis* and rice, respectively.

### 4.7. Phosphite Treatments

A stock solution of phosphite (KH_2_PO_3_, 1M) (Biosynth, Cymit Química S.L. Spain) was prepared in sterile water and stored at −20 °C until use. Working solutions of phosphate (Pi) plus phosphite (Phi) were freshly prepared (at 1/10th ratios of Pi relative to the corresponding Pi concentration). For the imaging of sensor plants, the Phi stock solution was added to half-strength Hoagland’s medium, pH 5.8 containing 0.1% agar, to the desired concentration. Transgenic sensor plants were subjected to the following treatments: 0.025 mM Pi + 0.0025 mM Phi; 0.25 mM Pi + 0.025 mM Phi; or 2.5 mM Pi + 0.25 mM Phi. Phosphite treatments are summarized in Table S2 and Table S3 for *Arabidopsis* and rice, respectively.

### 4.8. Live Imaging of Pi and FRET Analysis

For live Pi imaging, seedlings were placed on standard glass slides (75 × 26 mm, thickness ~1 mm), with a thin layer of 0.1% (*w*/*v*) agar and covered with No. 1 cover glasses (0.13–0.17 mm thickness; refractive index 1.515). The roots of the seedlings were immersed in 0.1% agar-based solution containing either control or treatment to keep the samples alive during imaging, as depicted in Figure S3. A coverslip was gently placed on top, leaving a gap between the coverslip and the slide to allow for the addition of Pi solutions or treatments during live imaging. During time-course experiments, buffered treatment solutions with 0.1% (*w*/*v*) agar base were applied to the slide, and the seedlings were maintained in a humid chamber to maintain ambient conditions uniformly for all samples.

Cytosolic Pi analysis in FLIPPi sensor plants is based on the Förster resonance energy transfer (FRET) between two fluorescent proteins, an enhanced cyan fluorescent protein (eCFP, donor) and a circularly permuted Venus (cpVenus, acceptor). The fluorescence of these FLIPPi lines was visualized by confocal laser scanning microscopy (CLSM). Upon Pi binding to the sensor’s phosphate-binding protein (PiBP) domain, a conformational change alters the distance and orientation between the fluorophores, resulting in a decrease in FRET emission. Thus, an increase in cytosolic Pi leads to a lower FRET signal, which can be quantified ratiometrically (cpVenus/eCFP intensity ratio). To improve measurement accuracy and reproducibility, the excitation and emission settings were optimized based on the spectral properties of eCFP and cpVenus. With 20% argon laser intensity, eCFP was excited at 458 nm, and its emission was collected at 483/32 nm. FRET-induced emission of cpVenus was recorded using the same 458 nm excitation but detected at 542/27 nm. Direct excitation of cpVenus was performed at 515 nm to assess acceptor fluorescence with emission at 542/27 nm. FRET images were acquired using a Leica SP5 confocal microscope (Leica Microsystems, Wetzlar, Germany) equipped with 20× dry objective lens.

For FRET analysis, imaging was focused on epidermal cells in the transition–elongation zone of primary roots, which represent key dynamic sites for nutrient sensing, environmental adaptation, and root development [34,35]. All confocal images were acquired as single optical sections (single focal planes) targeting this region. FRET analyses were performed using these single-plane images. Consistent imaging parameters, including laser power, detector gain, offset, pinhole size, and zoom factor, were maintained across all samples and experiments to ensure quantitative comparability.

The captured images were processed and analyzed using the ImageJ software (version 2.16.0/1.54p). Regions of interest were selected in root epidermal cells within transition and elongation zones, ensuring consistency across samples. FRET measurements were expressed as the ratio of cpVenus (acceptor) to eCFP (donor) intensity (cpVenus/eCFP), thus providing quantitative measures of Pi concentrations in plant roots.

### 4.9. Statistical Analysis

Data was calculated and analyzed for means, standard deviations, and standard errors using Microsoft Excel. Statistical significance was tested using one-way ANOVA and post hoc comparisons using Tukey’s HSD test, with the GraphPad Prism software (version 8.0.2) to assess the significance of differences between multiple treatment conditions and time-courses. Student’s *t*-test was used to evaluate statistical significance in sets of two data points.

## Data Availability

The original data presented in the study can be found within the published article and its Supporting Materials.

## Supplementary Materials

The following supporting information can be downloaded at https://www.mdpi.com/article/10.3390/plants14213334/s1. Figure S1. Validation of transgenic rice lines constitutively expressing either the cpFLIPPi5.3m biosensor or the cpFLIPPi-null sensor by RT-qPCR. Figure S2. Growth of cpFLIPPi5.3m *A. thaliana* and rice (*O. sativa* cv. Tainung 67) plants in response to Pi treatment. Figure S3. Live imaging setup for FRET analysis of *Arabidopsis* and rice roots. Table S1. Detailed summary of conditions and time-course treatments used for FRET experiments in *Arabidopsis*. Table S2. Detailed summary of conditions and time-course treatments used for FRET experiments in rice. Table S3. Oligonucleotides used for cloning of FLIPPi plasmids and expression analysis.

## References

[1] Lambers H. (2022). Phosphorus acquisition and utilization in plants. Annu. Rev. Plant Biol..

[2] Hinsinger P., Betencourt E., Bernard L., Brauman A., Plassard C., Shen J., Tang X., Zhang F. (2011). P for two, sharing a scarce resource: Soil phosphorus acquisition in the rhizosphere of inter-cropped species. Plant Physiol..

[3] Bucio J.S.L., González J.R., Bucio J.L. (2025). Adaptation of plants to phosphorus scarcity: From nutritional crosstalk to organellar function. Plant Sci..

[4] Péret B., Desnos T., Jost R., Kanno S., Berkowitz O., Nussaume L. (2014). Root architecture responses: In search of phosphate. Plant Physiol..

[5] Rouached H., Stefanovic A., Secco D., Bulak Arpat A., Gout E., Bligny R., Poirier Y. (2011). Uncoupling phosphate deficiency from its major effects on growth and transcriptome via PHO1 expression in *Arabidopsis*. Plant J..

[6] Paz-Ares J., Puga M.I., Rojas-Triana M., Martinez-Hevia I., Diaz S., Poza-Carrión C., Miñambres M., Leyva A. (2022). Plant adaptation to low phosphorus availability: Core signaling, crosstalks, and applied implications. Mol. Plant..

[7] Puga M.I., Poza-Carrión C., Martinez-Hevia I., Perez-Liens L., Paz-Ares J. (2024). Recent advances in research on phosphate starvation signaling in plants. J. Plant Res..

[8] Balzergue C., Chabaud M., Barker D.G., Bécard G., Rochange S.F. (2013). High phosphate reduces host ability to develop arbuscular mycorrhizal symbiosis without affecting root calcium spiking responses to the fungus. Front. Plant Sci..

[9] Campos-Soriano L., Bundó M., Bach-Pages M., Chiang S.F., Chiou T.J., San Segundo B. (2020). Phosphate excess increases susceptibility to pathogen infection in rice. Mol. Plant Pathol..

[10] Val-Torregrosa B., Bundó M., Martín-Cardoso H., Bach-Pages M., Chiou T.J., Flors V., Segundo B.S. (2022). Phosphate-induced resistance to pathogen infection in *Arabidopsis*. Plant J..

[11] Martín-Cardoso H., Bundó M., Val-Torregrosa B., Segundo B.S. (2024). Phosphate accumulation in rice leaves promotes fungal pathogenicity and represses host immune responses during pathogen infection. Front. Plant Sci..

[12] Martín-Cardoso H., Bücker G., Busturia I., San Segundo B. (2025). Unravelling mechanisms underlying phosphate-induced susceptibility to Bakanae disease in rice. Plant Stress..

[13] Val-Torregrosa B., Bundó M., Mallavarapu M.D., Chiou T.J., Flors V., San Segundo B. (2022). Loss-of-function of NITROGEN LIMITATION ADAPTATION confers disease resistance in *Arabidopsis* by modulating hormone signaling and camalexin content. Plant Sci..

[14] Danova-Alt R., Dijkema C., de Waard P., Köck M. (2008). Transport and compartmentation of phosphite in higher plant cells—Kinetic and P nuclear magnetic resonance studies. Plant Cell Environ..

[15] Jost R., Pharmawati M., Lapis-Gaza H.R., Rossig C., Berkowitz O., Lambers H., Finnegan P.M. (2015). Differentiating phosphate-dependent and phosphate-independent systemic phosphate-starvation response networks in *Arabidopsis* thaliana through the application of phosphite. J. Exp. Bot..

[16] Ticconi C.A., Delatorre C.A., Abel S. (2001). Attenuation of phosphate starvation responses by phosphite in *Arabidopsis*. Plant Physiol..

[17] Achary V.M.M., Ram B., Manna M., Datta D., Bhatt A., Reddy M.K., Agrawal P.K. (2017). Phosphite: A novel P fertilizer for weed management and pathogen control. Plant Biotechnol. J..

[18] Li Z., Kong X., Zhang Z., Tang F., Wang M., Zhao Y., Shi F. (2025). The functional mechanisms of phosphite and its applications in crop plants. Front. Plant Sci..

[19] Massoud K., Barchietto T., Le Rudulier T., Pallandre L., Didierlaurent L., Garmier M., Ambard-Bretteville F., Seng J.M., Saindrenan P. (2012). Dissecting phosphite-induced priming in *Arabidopsis* infected with *Hyaloperonospora arabidopsidis*. Plant Physiol..

[20] Piston D.W., Kremers G.J. (2007). Fluorescent protein FRET: The good, the bad and the ugly. Trends Biochem. Sci..

[21] Förster T. (1948). Zwischenmolekulare energiewanderung und fluoreszenz. Ann. Phys..

[22] Vogel S.S., van der Meer B.W., Blank P.S. (2014). Estimating the distance separating fluorescent protein FRET pairs. Methods.

[23] Mukherjee P., Banerjee S., Wheeler A., Ratliff L.A., Irigoyen S., Garcia L.R., Lockless S.W., Versaw W.K. (2015). Live imaging of inorganic phosphate in plants with cellular and subcellular resolution. Plant Physiol..

[24] Gu H., Lalonde S., Okumoto S., Looger L.L., Scharff-Poulsen A.M., Grossman A.R., Kossmann J., Jakobsen I., Frommer W.B. (2006). A novel analytical method for in vivo phosphate tracking. FEBS Lett..

[25] Choi G., Cha Y., Kim T.J., Lim G.H. (2025). Optimization of FRET imaging in *Arabidopsis* protoplasts. Mol. Cells..

[26] Banerjee S., Garcia L.R., Versaw W.K. (2016). Quantitative imaging of FRET-based biosensors for cell- and organelle-specific analyses in plants. Microsc. Microanal..

[27] Assunção A.G.L., Gjetting S.K., Hansen M., Fuglsang A.T., Schulz A. (2020). Live imaging of phosphate levels in *Arabidopsis* root cells expressing a FRET-based phosphate sensor. Plants.

[28] Ma L.J., Geiser D.M., Proctor R.H., Rooney A.P., O’Donnell K., Trail F., Gardiner D.M., Manners J.M., Kazan K. (2013). *Fusarium* pathogenomics. Annu. Rev. Microbiol..

[29] Plaxton W.C., Tran H.T. (2011). Metabolic adaptations of phosphate-starved plants. Plant Physiol..

[30] Chan C., Liao Y.Y., Chiou T.J. (2021). The Impact of Phosphorus on Plant Immunity. Plant Cell Physiol..

[31] Pérez-Zavala F.G., Ojeda-Rivera J.O., Herrera-Estrella L., López-Arredondo D. (2024). Beneficial effects of phosphite in *Arabidopsis thaliana* mediated by activation of ABA, SA, and JA biosynthesis and signaling pathways. Plants.

[32] Ames B.N. (1966). Assay of inorganic phosphate, total phosphate and phosphatases. Methods Enzymol..

[33] Versaw W.K., Harrison M.J. (2002). A chloroplast phosphate transporter, PHT2;1, influences allocation of phosphate within the plant and phosphate-starvation responses. Plant Cell.

[34] Bhosale R., Giri J., Pandey B.K., Giehl R.F.H., Hartmann A., Traini R., Truskina J., Leftley N., Hanlon M., Swarup K. (2018). A mechanistic framework for auxin-dependent *Arabidopsis* root hair elongation to low external phosphate. Nat. Commun..

[35] Serre N.B.C., Wernerová D., Vittal P., Dubey S.M., Medvecká E., Jelínková A., Petrášek J., Grossmann G., Fendrych M. (2023). The AUX1-AFB1-CNGC14 module establishes a longitudinal root surface pH profile. eLife.

