# Supplementary material for: Understanding Crosstalk Between Phosphate and Immune-Related Signaling in Rice and Arabidopsis Through Live Imaging of Phosphate Levels
Source: Plants (Basel). 2025 Oct 31;14(21):3334. doi: 10.3390/plants14213334 (PMC12610849; doi:10.3390/plants14213334)
Supplement: Supplementary file 1 [file plants-14-03334-s001.zip › plants-3938616-Supplementary Materials.pdf]

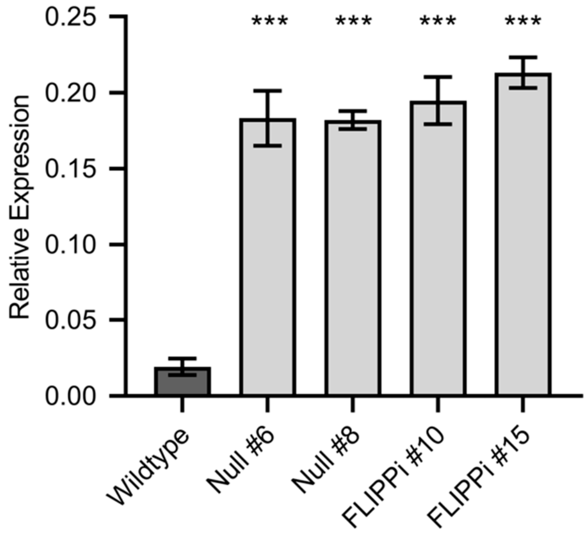

Figure S1. Validation of transgenic rice lines constitutively expressing either the cpFLIPPi5.3m biosensor or the cpFLIPPi-null sensor by RT-qPCR. Relative expression of *PiBP* gene of the FLIPPi sensor normalised to rice *Ubiquitin1* (*Ubi1*) gene in the obtained T_3 lines of cpFLIPPi5.3m, cpFLIPPi-null, or wildtype in *Oryza sativa* cv. Tainung 67 background. Statistical significance by Student's t-test, compared to control (wildtype), *** $p \leq 0.001$.

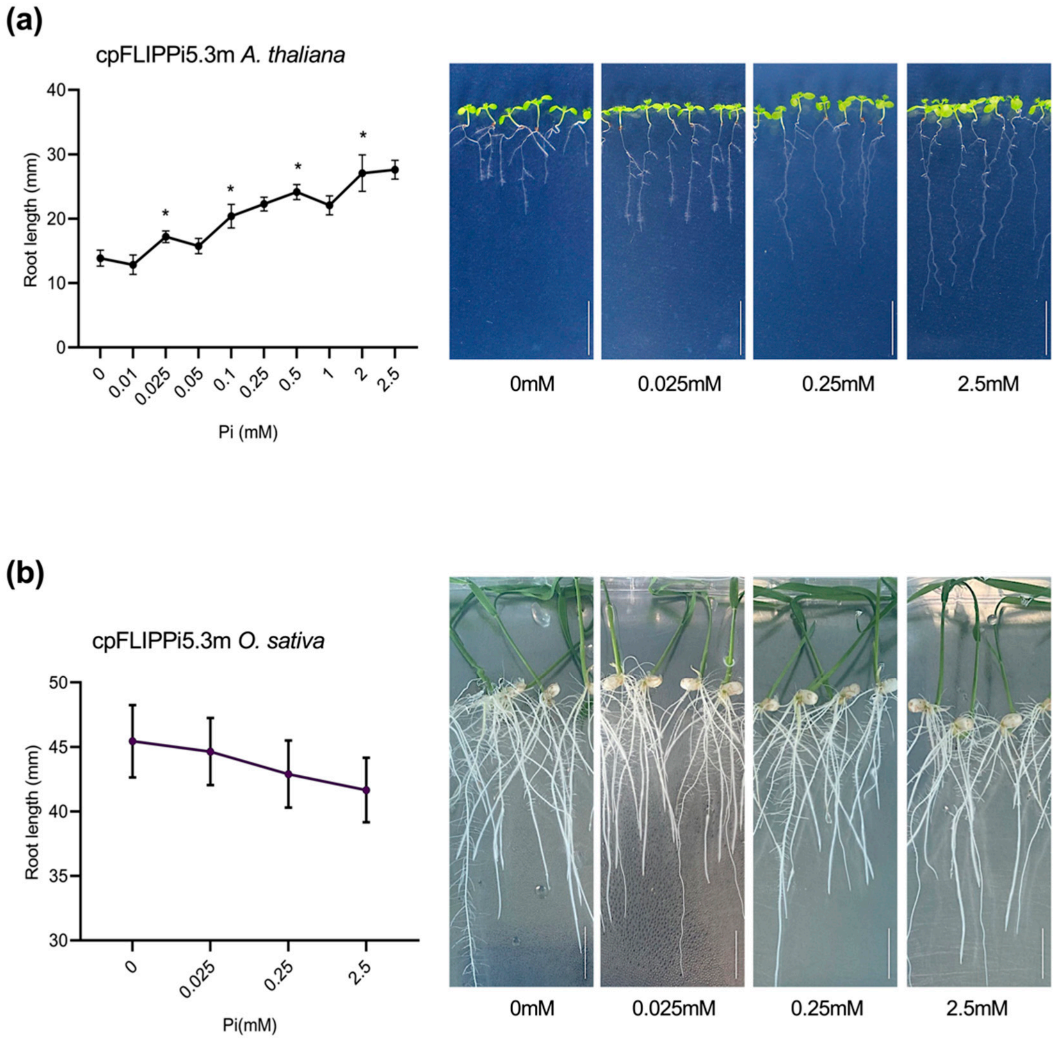

Figure S2. Growth of cpFLIPPi5.3m *A. thaliana* and rice (*O. sativa* cv. Tainung 67) plants in response to Pi treatment. **(a)** Average root length of cpFLIPPi5.3m *Arabidopsis*. Plants were grown for 1 week in half-strength MS media, and 1 week more in modified Hoagland's media with the indicated Pi concentrations. Right panel shows representative images of *Arabidopsis* plants at the indicated Pi concentrations. Scale bars: 10mm. **(b)** Average root length of cpFLIPPi5.3m rice. Rice seedlings were grown for 1 week in half-strength MS media, and 3 days more in modified Hoagland's media with increasing Pi concentrations. Right panel shows representative images of these rice roots. Scale bars: 10mm. Two independent experiments were carried out with at least 10 seedlings per condition; n ≥ 24). Bars represent Standard Error Mean (±SEM) compared to preceding sample, according to Student's *t*-test (* $p \leq 0.05$).

Table S1. Detailed summary of conditions and time-course treatments used for FRET experiments in *Arabidopsis*.

For Pi treatments: *Arabidopsis* seedlings were grown for first 7 days in ½ MS to synchronize all plants and then next 7 days conditioned in modified Hoagland media with different concentrations of Pi (0.025 mM Pi, 0.25 mM Pi and 2.5 mM Pi referred to as P_0, $P_{0.025}$, $P_{0.25}$ and $P_{2.5}$ respectively).
The 7-day treatment period used for *Arabidopsis* was to allow time, so the plants grow and acclimatize to different amounts of Pi, which helps us measure and get reproducible pattern of FRET ratios.

For Phi treatments: 7-days-old *Arabidopsis* seedlings were conditioned in media with Phi at 1/10th molar ratio of the corresponding Pi concentration, for 7 more days. The following conditions were tested (Figure 6c,d):

- Low Pi (0.025 mM Pi); Low Pi + Phi (0.025 mM Pi + 0.0025 mM Phi)
- Sufficient Pi (0.25 mM Pi); Sufficient Pi + Phi (0.25 mM Pi + 0.025 mM Phi)
- High Pi (2.5 mM Pi); High Pi + Phi (2.5 mM Pi + 0.25 mM Phi)

For time-course experiments: 2-weeks-old *Arabidopsis* seedlings were put on slides with treatment solution in 0.1% agar base with the following compositions:

Arabidopsis thaliana	Conditioned for 7 days in:	Solution applied for time-course		Results reference
		Control	Treatment	
Pi time-course	0.25 mM Pi	-	2.5 mM Pi	Figure 2e
Hormone time-course	0.25 mM Pi	0.25 mM Pi	0.25 mM Pi + 10 μM hormone	Figure 4a
Elicitor time-course	0.25 mM Pi	0.25 mM Pi	0.25 mM Pi + 100 μg/ml elicitor	Figure 4b
Phi time-course	0.025 mM Pi	0.025 mM Pi	0.025 mM Pi + 0.0025 mM Phi	Figure 6a

Table S2. Detailed summary of conditions and time-course treatments used for FRET experiments in rice.

For Pi treatments: Rice seedlings were grown for first 7 days in ½ MS to bring all plants to a conventional level of Pi and then 3 days of conditioning in modified Hoagland media with different concentrations of Pi (0.025 mM Pi, 0.25 mM Pi and 2.5 mM Pi denoted as P_0, $P_{0.025}$, $P_{0.25}$ and $P_{2.5}$ respectively). The 3-day treatment period used for rice is conventional and sufficient to detect distinct Pi levels and FRET ratios in the young root tissues for live-imaging.

For Phi treatments: 7-days-old rice seedlings were conditioned in media with Phi at $1/10^{th}$ molar ratio of the corresponding Pi concentration, for 3 days. The following conditions were tested (Figure 7c,d):

- Low Pi (0.025 mM Pi); Low Pi + Phi (0.025 mM Pi + 0.0025 mM Phi)
- Sufficient Pi (0.25 mM Pi); Sufficient Pi + Phi (0.25 mM Pi + 0.025 mM Phi)
- High Pi (2.5 mM Pi); High Pi + Phi (2.5 mM Pi + 0.25 mM Phi)

For time-course experiments: 10-days-old rice seedlings were put on slides with treatment solution in 0.1% agar base with the following compositions:

Oryza sativa	Conditioned for 3 days in:	Solution applied for time-course		Results reference
		Control	Treatment	
Pi time-course	0.25 mM Pi	-	2.5 mM Pi	Figure 3e
Hormone time-course	0.25 mM Pi	0.25 mM Pi	0.25 mM Pi + 10 µM hormone	Figure 5a
Elicitor time-course	0.25 mM Pi	0.25 mM Pi	0.25 mM Pi + 100 µg/ml elicitor	Figure 5b
Phi time-course	0.025 mM Pi	0.025 mM Pi	0.025 mM Pi + 0.0025 mM Phi	Figure 7a

(a)

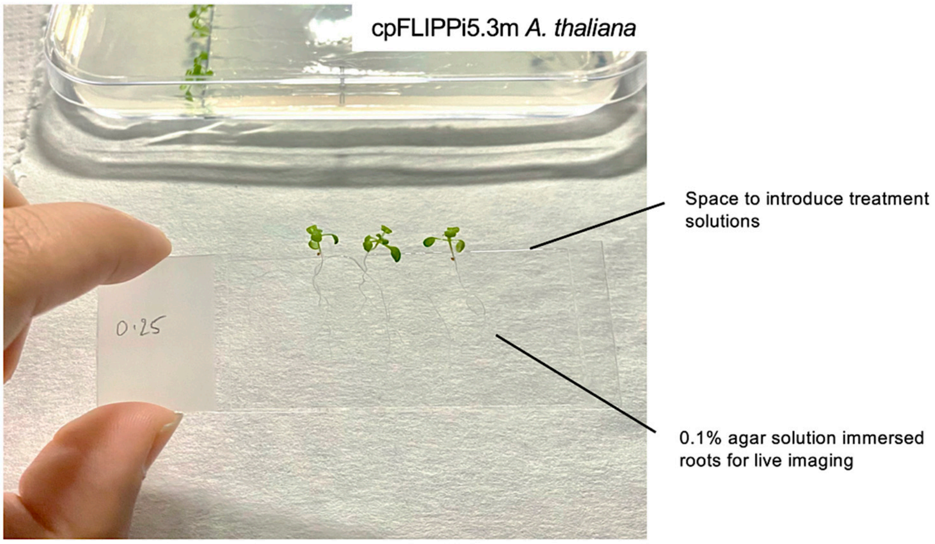

(b)

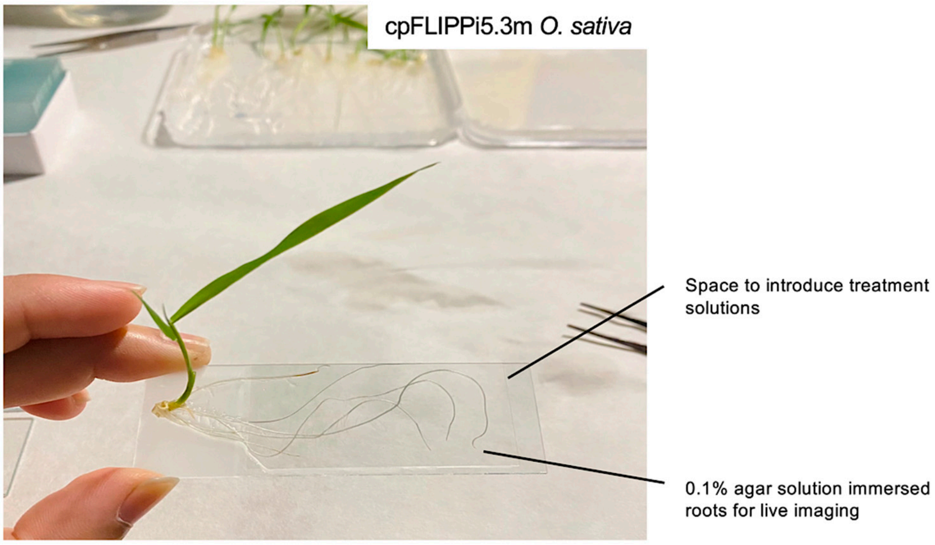

Figure S3. Live imaging setup for FRET analysis of *Arabidopsis* and rice roots. Seedlings were carefully positioned over the slides with the shoots above the slide and the roots immersed in 0.1% agar based solution, as either control or treatment. **(a)** Representative setup showing the placement of two-week-old *Arabidopsis thaliana* cpFLIPPi5.3m seedlings. **(b)** Placement of ten-day-old *Oryza sativa* cpFLIPPi5.3m seedlings on glass slides for live-cell FRET imaging.

Table S3. Oligonucleotides used for cloning of FLIPPi plasmids and expression analysis. Denoted as forward primer (F) and reverse primer (R).

Component	Organism	Gene ID	Purpose		Sequence 5′ → 3′
Ubi10 promoter	*Arabidopsis thaliana*	AT4g05320	Cloning & PCR	F	TGGTGTTAGTTTCTAGTTTGTGCG
NOS terminator	*Agrobacteria tumefaciens*	V00087.1	Cloning & PCR	R	TAATCATCGCAAGACCGGGCA
eCFP	*Aequorea victoria*	JX472997	Cloning & PCR	F	TGAGCAAAGACCTAAACGCTCT
cpVenus	*Aequorea victoria*	JX472996	Cloning & PCR	R	AGCTCAGGTAGTGGTTGTCG
Ubi1 promoter	*Oryza sativa*	LOC_Os06g46770	RT-qPCR	F	TTCCCCAATGGAGCTATGGTT
				R	AAACGGGACACGACCAAGG
PiBP	*Escherichia coli*	(Gene Index: 157829674)	Cloning & PCR	F	GCCGTCCATGTCGACGTTA
				R	CCGGGCAGGTTATAGGCAAA
			RT-qPCR	F	GACTACCCAATCGTGGGCTT
				R	GTGATGGCTTGACCCTCTGG
